# Generation of Monoclonal Antibodies against Immunoglobulin Proteins of the Domestic Ferret (*Mustela putorius furo*)

**DOI:** 10.1155/2017/5874572

**Published:** 2017-02-14

**Authors:** Greg A. Kirchenbaum, Ted M. Ross

**Affiliations:** ^1^Center for Vaccines and Immunology, University of Georgia, Athens, GA, USA; ^2^Department of Infectious Diseases, University of Georgia, Athens, GA, USA

## Abstract

The domestic ferret (*Mustela putorius furo*) serves as an animal model for the study of several viruses that cause human disease, most notably influenza. Despite the importance of this animal model, characterization of the immune response by flow cytometry (FCM) is severely hampered due to the limited number of commercially available reagents. To begin to address this unmet need and to facilitate more in-depth study of ferret B cells including the identification of antibody-secreting cells, eight unique murine monoclonal antibodies (mAb) with specificity for ferret immunoglobulin (Ig) were generated using conventional B cell hybridoma technology. These mAb were screened for reactivity against ferret peripheral blood mononuclear cells by FCM and demonstrate specificity for CD79*β*^+^ B cells. Several of these mAb are specific for the light chain of surface B cell receptor (BCR) and enable segregation of kappa and lambda B cells. Additionally, a mAb that yielded surface staining of nearly all surface BCR positive cells (*i.e.*, pan ferret Ig) was generated. Collectively, these M*α*F-Ig mAb offer advancement compared to the existing portfolio of polyclonal anti-ferret Ig detection reagents and should be applicable to a wide array of immunologic assays including the identification of antibody-secreting cells by FCM.

## 1. Introduction

The domestic ferret (*Mustela putorius furo*) serves as an animal model for the study of several viruses that cause human disease [1, 2]. Most notably, ferrets are naturally susceptible to human influenza virus and are capable of viral transmission [3–6]. Their application to influenza research began in 1933, when throat washings from human subjects were administered intranasally into ferrets [6]. These animals went on to exhibit outward symptoms of influenza including fever, sneezing, and lethargy. Transmission of influenza-like disease was also observed following transfer of nasal washings from an infected ferret or cohousing with a naïve contact. Due to the expression of both *α*2,6 and *α*2,3 sialic acid moieties along the respiratory tract, ferrets are permissive to infection with human seasonal and prepandemic avian influenza isolates [7]. Moreover, they recapitulate the extrapulmonary replication of highly virulent avian influenza subtypes such as H5N1 and H7N7 [8, 9]. Collectively, the ferret model has provided invaluable insights into the antigenicity, virulence and transmissibility of circulating and newly emerging influenza isolates [1, 10–12].

Despite the usefulness of the ferret animal model, the lack of ferret-specific reagents has severely hampered the ability to perform in-depth immunologic profiling. Recent studies have implemented more detailed methods for interrogating the immune response elicited in the ferret, including quantitative RT-PCR to measure cytokine and chemokine transcript levels and flow cytometric analysis of leukocyte populations utilizing cross-reactive monoclonal antibody (mAb) reagents [13–17]. However, mAb with defined specificity for B cell lineage surface markers, such as CD19 [18], that would facilitate identification of ferret B cells at various developmental stages by flow cytometry (FCM) are currently lacking. To circumvent this issue, others have identified ferret B cells on the basis of surface immunoglobulin (Ig) and/or CD79*α* expression [14, 16]. While CD79*α* is an excellent marker for identification of B lineage cells because it is an obligate chaperone for surface expression of B cell receptor (BCR) [19], the epitope recognized by this mAb (clone HM47) requires intracellular staining and thus does not permit isolation of viable B cells [20]. Alternatively, polyclonal antibodies with reactivity against ferret Ig are conducive for surface staining but do not exclusively define B cells due to binding of ferret Ig by myeloid cells via Fc receptors [14]. Moreover, neither approach enables segregation of ferret B cells on the basis of heavy chain usage. Collectively, the suite of available reagents is still insufficient for an in-depth characterization of the humoral immune response, and specifically the identification of antibody-secreting cells by FCM. To begin to address this unmet need, several novel mAb with specificity for ferret immunoglobulin (Ig) were generated and characterized to define their specificity.

## 2. Materials and Methods

### 2.1. Animals

BALB/c mice (female, 8–10 weeks of age) from Jackson Laboratory (Bar Harbor, ME, USA) and Fitch ferrets (*Mustela putorius furo*, male or female, 6 to 12 months of age) from Triple F Farms (Sayre, PA, USA) were housed in cage units and fed ad libitum. All animals were handled in accordance with protocols approved by Institutional Animal Care and Use Committees and were cared for under USDA guidelines for laboratory animals.

### 2.2. Isolation of Ferret Peripheral Mononuclear Cells

Peripheral blood was collected via cardiac puncture into vacuum collection tubes containing sodium heparin (Becton Dickinson, Cat #367874) and gently inverted to prevent coagulation. Blood was then combined with phosphate buffered saline (PBS) (Corning, Cat #21-040-CV) to a final volume of 35 mL and overlaid on 10 mL Ficoll-Paque PLUS (GE Healthcare, Cat #17-1440) before centrifugation at 500 ×g for 25 min with the brake reduced to its lowest setting using a Sorvall Legend XTR (Thermo Scientific, Grand Island, NY, USA). Peripheral blood mononuclear cells (PBMC) at the interface were collected, washed with PBS, and then pelleted (400 ×g for 10 min). Following an additional wash with PBS, total cell number and viability was determined by Trypan blue exclusion using the Countess™ (ThermoFisher, Cat #C10227). Ferret PBMC were used immediately or resuspended in fetal bovine serum (FBS) (HyClone, Cat #SH30396.03) containing 10% DMSO (Thermo Scientific, Cat #20688) for long-term storage. Aliquots of 1-2 × 10^7^ viable ferret PBMC were stored in the vapor phase of liquid nitrogen until use and thawing of cells was according to similarly described methods [22].

### 2.3. Purification of Ferret Immunoglobulin

Serum from two ferrets (female, 7 months of age) was pooled and immunoglobulin (Ig) precipitated by drop-wise addition of an equal volume of saturated ammonium sulfate solution (4.1 M) (Sigma, Cat #A4418) while maintaining the solution under constant agitation at 4°C. Precipitated protein was pelleted by centrifugation at 11,5000 rpm for 20 min at 4°C and then dissolved in PBS. The protein solution was then transferred into a Slide-A-Lyzer dialysis cassette (ThermoFisher, Cat #66030) and dialyzed against PBS at 4°C for three days with daily buffer exchanges. Subsequently, the protein solution was clarified by centrifugation at 6000 rpm for 10 min at 4°C and then passed through a 0.2 *µ*m syringe filter (ThermoFisher, Cat #09-719C). This material is referred to as crude ferret Ig. Ferret Ig was further purified by affinity chromatography using Protein A/G (ThermoFisher, Cat #20423). Briefly, crude ferret Ig protein solution was applied to Protein A/G and the column (Bio-Rad, Cat #7311550) was washed by gravity flow with PBS. Fractions (2 mL) were collected and absorbance (280 nm) was monitored using a PowerWave XS spectrometer (Biotek, Winooski, VT, USA). Once wash fractions returned to baseline, ferret Ig protein was eluted by addition of 0.1 M glycine, pH 2.5 (Amresco, Cat #M103). Eluted protein was immediately neutralized with 200 *µ*l 1.5 M Tris, pH 8.8 (Amresco, Cat #M151) and protein containing elution fractions were pooled, buffer exchanged into PBS containing 0.05% sodium azide (Sigma, Cat #S2002), and concentrated using a Spin-X UF filter (Sigma, Cat #CLS431489). This material is referred to as purified ferret Ig. Protein concentrations of the crude and purified ferret Ig solutions were determined according to the manufacture's instructions using a micro BCA™ assay kit (ThermoFisher, Cat #23235).

### 2.4. Protein Gel Electrophoresis

To assess purity of the respective ferret Ig containing protein solutions, 5 *µ*g of crude or purified ferret Ig was loaded into Bolt™ 10% Bis-Tris Plus precast protein gels (ThermoFisher, Cat #NW00102) and resolved at 150 V for 50 min. Protein samples were diluted in either 2x Laemmli sample buffer (Bio-Rad, Cat #161-0737) with or without *β*-mercaptoethanol (JT Baker, Cat #P62405) or 6x SDS-sample buffer (reducing) (Boston BioProducts, Cat #BP-111R). Reduced samples were heated at 100°C for 10 min and placed on ice prior to loading. Gels were stained with PageBlue™ protein staining solution (ThermoFisher, Cat #24620) and then destained in water before imaging using the myECL Imager (ThermoFisher, Waltham, MA, USA). Spectra™ Multicolor Broad Range Protein Ladder (ThermoFisher, Cat #26634) was included in all gels and used for molecular marker reference.

### 2.5. Generation of Murine Monoclonal Antibodies

Female BALB/c mice were immunized with 100 *µ*g of purified ferret Ig containing the Imject alum adjuvant (Thermo Scientific, Cat #77161) via the intraperitoneal route. Serum was collected via the submandibular vein on day 21 and assessed by ELISA for antibody reactivity (refer to ELISA subsection). The two mice with the highest antibody titer received a booster immunization, via the intraperitoneal route, containing 100 *µ*g of purified ferret Ig in PBS on Day 28. Three or four days after the booster immunization, splenocytes were harvested and used to perform a fusion with the SP2/O myeloma (kindly provided by Dr. Lawrence Wysocki, University of Colorado Denver) using polyethylene glycol 1450 (ATCC, Cat #50-X). Hybridomas were selected by addition of hypoxanthine (Acros Organics, Cat #1220100) and azaserine (Sigma, Cat #A4142) at a final concentration of 200 *µ*M and 11.5 *µ*M respectively in RPMI 1640 (Sigma, Cat #R6504) containing 10% FBS, 23.8 mM sodium bicarbonate (Fisher Scientific, Cat #BP328), 7.5 mM HEPES (Amresco, Cat #0485), 170 *µ*M Penicillin G (Tokyo Chemical Industry, Cat #P1770), 137 *µ*M Streptomycin (Sigma, Cat #S9137), 50 *µ*M *β*-mercaptoethanol, and 1 mM sodium pyruvate (ThermoFisher, Cat #11360070). Eleven days after the respective fusions, culture supernatants were screen for reactivity by ELISA (refer to ELISA subsection). Positive wells were further expanded and maintained under drug selection, and hybridoma cell lines of interest were subcloned by limiting dilution. Hybridomas were subsequently expanded in media containing 5% IgG-stripped FBS (Hyclone, Cat #SH30898.03) and monoclonal antibody (mAb) purified by affinity chromatography (Protein A/G) as described above.

### 2.6. ELISA

The enzyme-linked immunosorbent assay (ELISA) was used to assess antibody reactivity against purified ferret Ig and to determine the IgG subclass and concentration of the respective mAb. To measure antibody binding against the purified ferret Ig antigen, CoStar high binding ELISA plates (Corning, Cat #3590) were coated overnight at 4°C with 2 *µ*g/mL purified ferret Ig in carbonate buffer pH 9.4 containing 5 *µ*g/mL fraction V bovine serum albumin (BSA) (Equitech-Bio, Cat #BAC69). Alternatively, plates were coated with 5 *µ*g BSA in carbonate buffer alone. Plates were blocked with ELISA block buffer, PBS containing 0.2% BSA, 0.1% bovine gelatin (Sigma, Cat #G9391), and 0.05% Tween 20 (Sigma, Cat #P7949), for 90 min at 37°C prior to addition of culture supernatant or antibody solutions. Culture supernatants were diluted 1 : 2 for hybridoma screening, and purified mAb were diluted to 3 *μ*g/mL in ELISA blocking buffer prior to 3-fold serial dilution. Plates were incubated for 90 min at 37°C and washed with PBS to remove unbound antibody. For hybridoma screening, horseradish peroxidase conjugated goat anti-mouse IgG (*γ*-specific) (Southern Biotech, Cat #1030-05) secondary antibody diluted in blocking buffer was added to ELISA plates. Alternatively, binding of mAb to ferret Ig was revealed by addition of horseradish peroxidase conjugated goat anti-mouse IgG1 (*γ*1-specific) (Southern Biotech, Cat #1070-05) secondary antibody. After addition of secondary antibody, plates were incubated for 60 min at 37°C and then washed extensively with PBS prior to addition of 2,2-azino-bis (3-ethylbenzothiazoline-6-sulfonic acid) (ABTS) (Amresco, Cat #0400) substrate. Plates were incubated at 37°C for development and colorimetric conversion was terminated by addition of 5% sodium dodecyl sulfate (SDS) (Teknova, Cat #S0294). Optical density was measured at 414 nm (OD_414_) using a PowerWave XS spectrophotometer.

To determine the IgG subclass or determine the concentration of the respective mAb, CoStar high binding ELISA plates were coated overnight at 4°C with 1 *μ*g/mL goat anti-mouse IgG (*γ*-specific) capture antibody (Sigma, Cat #M1397) in carbonate buffer pH 9.4 containing 5 *μ*g/mL BSA. Plates were then blocked with ELISA blocking buffer for 90 min at 37°C. Culture supernatants from clonal hybridoma lines of interest, diluted mouse anti-ferret Ig (M*α*F-Ig) mAb or mouse IgG1,*κ* (Biolegend, Cat #401402), were serially diluted in ELISA blocking buffer and plates incubated for 90 min at 37°C. Plates were washed five times with PBS, horseradish peroxidase conjugated goat anti-mouse IgG1 (*γ*1-specific) secondary antibody diluted in ELISA blocking buffer added and the plates incubated for 60 min at 37°C. Following extensive washing with PBS, ABTS substrate was added and plates incubated at 37°C for development. Colorimetric conversion was terminated by addition of 5% SDS solution, and OD_414_ was measured using a PowerWave XS spectrophotometer. The concentration of individual mAb was then interpolated based on a nonlinear regression of the IgG1,*κ* standard using PRISM 6.0 (GraphPad Software, La Jolla CA, USA).

### 2.7. Competitive ELISA

A competitive ELISA was performed using unlabeled and biotinylated M*α*F-Ig mAb (refer to Protein Conjugation) to identify overlapping epitope recognition. CoStar high binding ELISA plates were coated overnight at 4°C with 2 *µ*g/mL purified ferret Ig in carbonate buffer pH 9.4 containing 5 *µ*g/mL BSA. Plates were then blocked with ELISA blocking buffer for 90 min at 37°C. Unlabeled M*α*F-Ig mAb (5–50 *µ*g/mL starting concentration, determined using BCA assay kit) were serially diluted in ELISA blocking buffer, followed by addition of biotinylated M*α*F-Ig mAb, and plates incubated overnight at 4°C. Plates were washed five times with PBS, horseradish peroxidase conjugated streptavidin diluted in blocking buffer added, and the plates incubated for 60 min at 37°C. Following extensive washing with PBS, ABTS substrate was added and plates incubated at 37°C for development. Colorimetric conversion was terminated by addition of 5% SDS solution, and OD_414_ was measured using a PowerWave XS spectrophotometer. The percent of maximal signal was determined using the formula 100 × [OD_414_ experimental sample − OD_414_ blank/OD_414_ maximal signal − OD_414_ blank].

### 2.8. Western Blot

To characterize the specificity of individual M*α*F-Ig mAb, 1 *µ*g of purified ferret Ig or 0.5 *µ*l of ferret serum was reduced and resolved by protein gel electrophoresis as describe previously. Protein transfer to polyvinylidene difluoride (PVDF) membranes was performed using the Trans-Blot Turbo RTA Mini PVDF transfer kit (Bio-Rad, Cat #1704272) and a Trans-Blot Turbo Blotting system (Bio-Rad, Hercules, CA, USA) according to the manufacture's instructions. The membrane was blocked with PBS + Tween 20 (0.1% v/v) (PBST) containing 5% BSA (VWR, Cat #0332) at room temperature (RT) with constant agitation. The PVDF membranes were then cut into strips and probed with 15 mL of PBST containing 0.1 *µ*g/mL of individual M*α*F-Ig mAb overnight at RT. The following day, PVDF membranes were washed three times with PBST before incubation for 60 min at RT with 10 mL PBST containing horseradish peroxidase conjugated goat anti-mouse IgG1 (*γ*1-specific). Following extensive washing with PBS, membranes were treated with 4 mL Clarity™ Western ECL Substrate (Bio-Rad, Cat #1705060) and imaged using myECL Imager. Postacquisition analysis was performed using myImageAnalysis™ Software (ThermoFisher).

### 2.9. Flow Cytometry

To evaluate the specificity of commercially available anti-ferret Ig reagents by flow cytometry (FCM), ferret PBMC were labeled with FITC conjugated goat anti-ferret IgM (*µ*-specific) (Sigma, Cat # SAB3700807) or biotin conjugated goat anti-ferret IgG (G*α*F-Ig*γ* BIO) (Sigma, Cat #SAB3700796). Binding of G*α*F-Ig*γ* BIO was revealed using phycoerythrin conjugated streptavidin (SA-PE) (Biolegend, Cat #405204). The reactivity of individual mAb against ferret leukocytes was assessed by direct or indirect staining. Initially, culture supernatants from clonal hybridoma lines were diluted to 1 *µ*g/mL in FCM staining buffer (PBS + 2% FBS + 0.1% NaN_3_) and used to stain ferret PBMC. Because this screening method was dependent on revealing binding of the murine mAb using a polyclonal goat anti-mouse Ig Alexa Fluor 647 (G*α*M-Ig AF647) reagent (BD, Cat #51-9006588BK), we were unable to utilize a cross-reactive anti-CD79*β* murine mAb (clone CB3-1) to identify B cells [16, 23]. Ferret B cells were instead identified using G*α*F-Ig*γ* BIO, which was revealed using SA-PE. Binding of murine mAb to ferret PBMC was revealed with G*α*M-Ig AF647. All staining was performed on ice in 100 *µ*l of volume and cells were stained with LIVE/DEAD Aqua (ThermoFisher, Cat #L34957) prior to surface staining to enable exclusion of nonviable cells.

For direct staining, ferret PBMC were initially stained with LIVE/DEAD Aqua and then pretreated with irrelevant Mouse IgG2a,*κ* (Biolegend, Cat #401502) and Rat IgG2a,*κ* (Biolegend, Cat #400502) to exclude nonviable cells and minimize nonspecific binding. Ferret PBMC were then stained with anti-CD79*β* (Biolegend, Cat #341408) and DyLight 488, DyLight 650, and/or biotin conjugated M*α*F-Ig mAb (refer to Protein Conjugation). Biotinylated mAb were revealed by secondary staining with SA-PE. In order to putatively identify kappa light chain expressing B cells, ferret PBMC were stained with DyLight 650 conjugated recombinant Protein L (ThermoFisher, Cat # 21189) and anti-CD79*β* simultaneously with DyLight 488 and biotin conjugated M*α*F-Ig mAb. Acquisition was performed on the LSR II cytometer (BD Biosciences, San Jose, CA, USA) and analysis performed with FlowJo Version 10.0.8 (Tree Star, Ashland, OR, USA).

### 2.10. Protein Conjugation

Protein A/G purified mAb or recombinant Protein L were conjugated to DyLight 488 (ThermoFisher, Cat #46402), DyLight 650 (ThermoFisher, Cat #62265), or EZ-Link™ NHS-LC-Biotin (ThermoFisher, Cat #21336) according to the manufacture's instructions. Unreacted DyLight or NHS-LC-Biotin was removed by multiple buffer exchanges into PBS containing 0.05% sodium azide using Spin-X UF filters (30 kDa MWCO).

### 2.11. Sequencing Hybridoma V Region Genes

Variable genes of B cell hybridomas were cloned by RT-PCR and 5′ rapid amplification of cDNA ends (RACE) using pairs of constant region and anchor primers according to previously described methods, with minor modifications [24]. Briefly, total RNA was isolated from hybridoma lines using RNeasy (Qiagen, Cat #74104) and first-strand cDNA synthesis performed using SuperScript III RT (ThermoFisher, Cat #18080051) using oligo-dT primer. First-strand cDNA was isolated using QIAquik PCR spin columns (Qiagen, Cat #28104) and then dG-tailed with TdT (NEB, Cat #M0315S) and dGTP (ThermoFisher, Cat #10218014). Variable* IgH* or* Igκ* genes were then amplified from dG-tailed cDNA templates using Phusion (NEB, Cat# M0530S). A poly-A tail was added to products following completion of the second round of PCR by addition of 5 units recombinant Taq polymerase (ThermoFisher, Cat #EP0402) directly into the reaction and incubation at 72°C for 10 min. Products from* Igκ* PCR were further purified with QIAquick PCR spin columns before digestion with restriction enzymes PflFI (NEB, Cat #R0595S) or PflmI (NEB, Cat #R0509S) to disrupt the rearranged* Vκ21-12* gene from the SP2/0 fusion partner. After 2% agarose electrophoresis, the uncut V*κ* products were isolated using the QIAquik gel extraction kit (Qiagen, Cat #28704) and eluted with autoclaved water. Variable region genes were cloned into pCR4-TOPO (ThermoFisher, Cat #K4575J10) or pSC-A (Agilent, Cat #240205) plasmids according to the manufacture's instructions. Plasmid DNA was purified using QIAprep spin columns (Qiagen, Cat #27104) and submitted to Macrogen (Rockville, MD, USA) for sequencing. Heavy and kappa variable region genes were identified using IMGT V-Quest [21].

### 2.12. Statistics

Statistical analyses were performed using PRISM 6.0.

## 3. Results

### 3.1. Commercial Reagents against Ferret Immunoglobulin Lack Heavy Chain Specificity

Expression of a class-switched B cell receptor (BCR), such as IgG or IgA, can be used as a marker of memory B cells, while naïve B cells express an IgM BCR [25]. As a first attempt to segregate ferret B cells into naïve and memory compartments on the basis of surface BCR expression, ferret PBMC were stained with polyclonal goat anti-ferret IgM (G*α*F-Ig*µ*) or goat anti-ferret IgG (G*α*F-Ig*γ*) antiserum. Additionally, the mouse anti-human CD79*β* mAb (clone CB3-1), which cross-reacts with ferret leukocytes (Supplementary Materials available online at https://doi.org/10.1155/2017/5874572), was included in the staining solution to identify surface BCR^+^ cells [16]. The G*α*F-Ig*µ* antisera labeled ~99% of the CD79*β*^+^ population, while the G*α*F-Ig*γ* antisera costained ~66% of the CD79*β*^+^ population (Supplementary Materials). To extend these observations, ferret PBMC were next stained with both the G*α*F-Ig*µ* and G*α*F-Ig*γ* simultaneously. The majority of CD79*β*^+^ ferret PBMC exhibited staining with both the G*α*F-Ig*µ* and G*α*F-Ig*γ* reagents. Collectively, these findings indicate that surface staining with anti-CD79*β* enables identification of ferret B cells and currently available anti-ferret Ig reagents are insufficient to discriminate B cells on the basis of heavy chain expression.

### 3.2. Purification of Ferret Immunoglobulin

**Figure 1:**
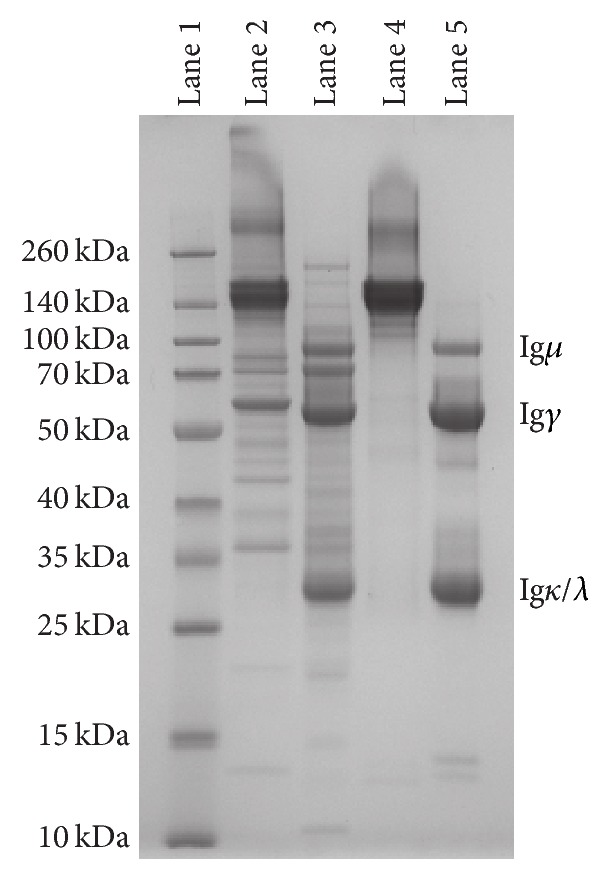
SDS-polyacrylamide gel electrophoresis of purified ferret immunoglobulin. 5 *µ*g of crude (lanes 2 and 3) or Protein A/G purified (lanes 4 and 5) ferret Ig preparations were resolved by SDS-PAGE under nonreducing (lanes 2 and 4) or reducing (lanes 3 and 5) conditions. Molecular marker references are indicated (lane 1). Image was acquired using the myECL imager and contrast adjustment of the entire image was performed using myImageAnalysis software. Image is representative of two independent purifications.

Ferret Ig was first crudely enriched from serum through ammonium sulfate precipitation and the resulting protein solution was predominantly IgG (Figure 1, lanes 2 and 3). Next, ferret Ig was further purified by affinity chromatography using Protein A/G. This second purification step removed the majority of contaminating proteins and produced a highly pure ferret Ig preparation (Figure 1, lanes 4 and 5). Reduction of the purified ferret Ig into heavy and light chain components confirmed the presence of both Ig*µ* and Ig*γ* on the basis of their differential sizes (Figure 1, lane 5) [26].

### 3.3. Immunization with Purified Ferret Immunoglobulin

Mouse IgG2a mAb have increased nonspecific binding to ferret leukocytes relative to IgG1 (data not shown). In order to elicit an antibody response utilizing the IgG1 subclass, BALB/c mice were immunized with purified ferret Ig prepared with the Imject alum adjuvant [27]. Following a single immunization with ferret Ig and adjuvant, antigen-specific reactivity was detected by ELISA and western blot (Supplementary Materials). Moreover, reduction of ferret Ig in the SDS-PAGE enabled discrimination between antibody reactivity with the light (Ig*κ*/Ig*λ*) and heavy (Ig*µ* or Ig*γ*) chain components of ferret Ig.

### 3.4. Characterization of Monoclonal Antibodies by ELISA

**Figure 2:**
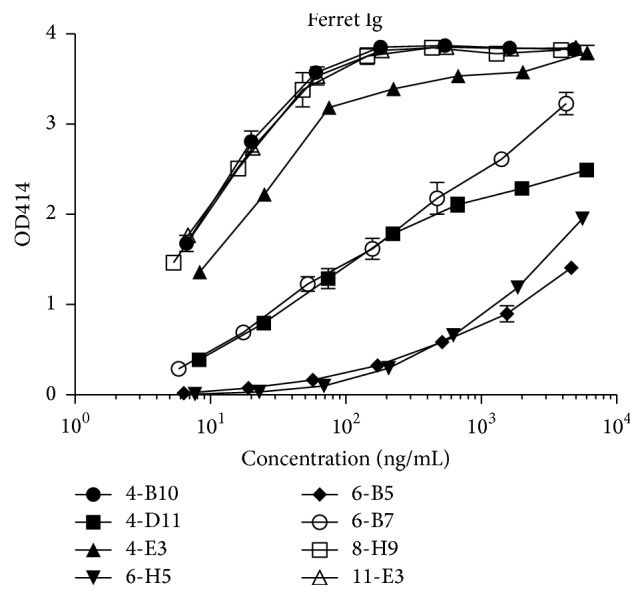
Monoclonal antibody binding to ferret immunoglobulin. Mouse mAb were evaluated for binding to purified ferret Ig by ELISA. Optical densities are plotted versus normalized IgG1 input (*x*-axis). Data are representative of two or more independent experiments that yielded similar results.

Based on their reactivity with ferret Ig, two mice (#3 and #5) were chosen for mAb generation. Eight IgG1^+^ mAb, collected from two independent fusions, reacted with purified ferret Ig. Each of these mouse anti-ferret Ig (M*α*F-Ig) mAb reacted with ferret Ig by ELISA (Figure 2). Additionally, normalization of the input IgG1 concentration highlighted the distinct binding curves of several M*α*F-Ig mAb.

### 3.5. Assessment of Monoclonal Antibody Reactivity by Western Blot

**Figure 3:**
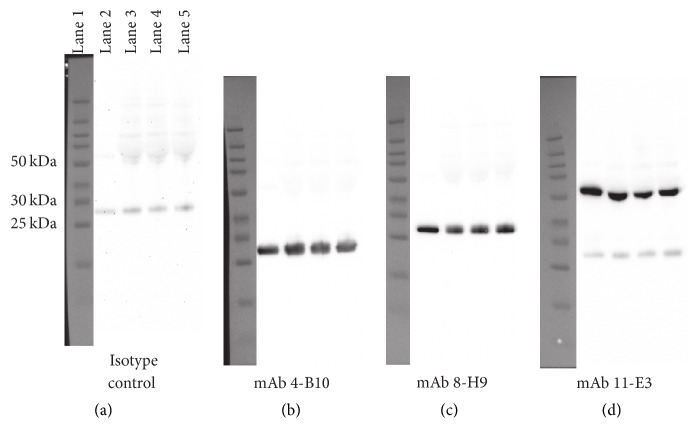
Reactivity of monoclonal antibody against reduced ferret immunoglobulin. 1 *µ*g of ferret Ig (lane 2) or 0.5 *µ*l of ferret serum (*n* = 3) (lanes 3–5) was resolved by SDS-PAGE under reducing conditions and transferred to PVDF membranes. Membranes were then cut into strips and probed with (a) irrelevant IgG1, (b)* 4-B10*, (c)* 8-H9*, or (d)* 11-E3*. Images were acquired using the myECL imager and contrast adjustments of the entire image were performed using myImageAnalysis software. Visible light images of the molecular markers (lane 1) are presented next to the corresponding chemiluminescent image for reference. Images are representative of two independent experiments.

As an additional technique to further characterize the reactivity of the respective mAb with ferret Ig, individual M*α*F-Ig mAb were used to probe reduced ferret Ig antigen and serum from three naïve ferrets in a western blot screen. Three of the eight M*α*F-Ig mAb demonstrated specific reactivity with ferret Ig antigen using this assay (Figure 3). Both M*α*F-Ig mAb* 4-B10* and* 8-H9* reacted with ferret Ig light chain protein (Figures 3(b) and 3(c)). Additionally, mAb* 11-E3* reacted with a protein species corresponding to the Ig*γ* chain (Figure 3(d)). Of note, these M*α*F-Ig mAb reacted with both purified ferret Ig antigen and serum samples, suggesting the epitopes recognized by the respective mAb are not polymorphic.

### 3.6. Assessment of Monoclonal Antibody Reactivity by Flow Cytometry

**Figure 4:**
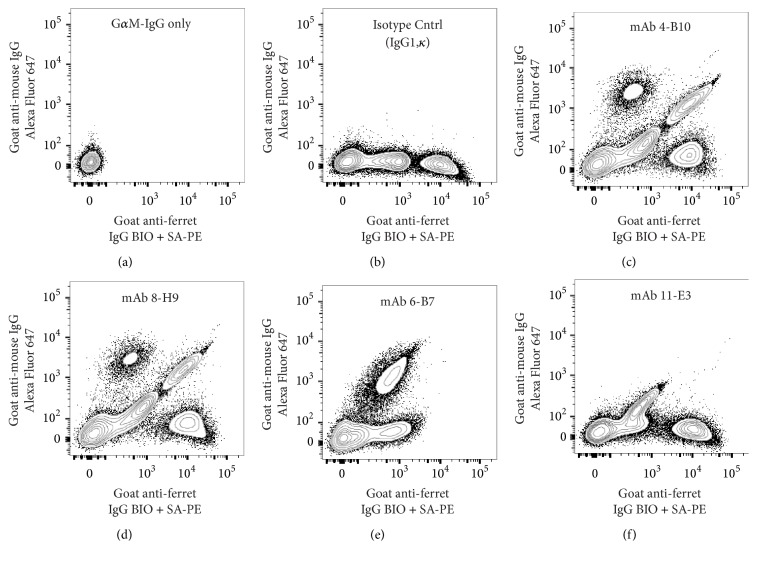
Flow cytometric assessment of monoclonal antibody reactivity. Reactivity of mouse anti-ferret Ig mAb with ferret PBMC was evaluated by flow cytometry. Binding of mouse Ig to ferret PBMC was revealed by secondary staining with Alexa Fluor 647 conjugated goat anti-mouse IgG (G*α*M-IgG). Additionally, ferret B cells were identified by costaining with biotinylated goat anti-ferret IgG, which was revealed by secondary staining with phycoerythrin conjugated streptavidin (SA-PE). (a) Reactivity of the G*α*M-IgG secondary antibody with ferret PBMC. (b–f) 1 *µ*g of an IgG1 control (b) or mouse anti-ferret Ig mAb (c–f) was used for indirect surface staining of ferret PBMC. The presented data were generated using PBMC from a single ferret and are representative of two or more independent experiments that yielded similar results.

In spite of earlier observations suggesting that the G*α*F-Ig*γ* antisera was unlikely to define IgG^+^ ferret B cells exclusively (Supplementary Materials), this reagent was incorporated into a flow cytometric screening assay to evaluate the ability of each M*α*F-Ig mAb to bind ferret PBMC because it enabled resolution of these cells into distinct populations on the basis of staining intensity (Figure 4(b)). The two populations of ferret PBMC that demonstrated staining with the G*α*F-Ig*γ* antisera are referred to as IgG^int^ and IgG^hi^, respectively, for the sole purpose of detailing the staining patterns observed for the respective M*α*F-Ig mAb (Figure 4 and data not shown). Using this flow cytometric screening approach, both* 4-B10* and* 8-H9* had similar staining patterns of ferret PBMC and labeled ~50% of the IgG^hi^ population (Figures 4(c) and 4(d)). Moreover, both of these M*α*F-Ig mAb also reacted with a small population of IgG^int^ cells. Three additional M*α*F-Ig mAb (*4-E3*,* 4-D11*, and* 6-H5*) had similar reactivity patterns (data not shown). A distinct staining pattern was observed using two M*α*F-Ig mAb (*6-B5* and* 6-B7*) (Figure 4(e) and data not shown). Specifically, pretreatment of ferret PBMC with these mAb resulted in the disappearance of the IgG^hi^ population, likely due to BCR internalization or blocking of epitopes targeted by the G*α*F-Ig*γ* antisera. Additionally, a subset of the IgG^int^ population was labeled by* 6-B7* on the basis of G*α*F-Ig*γ* staining (Figure 4(e)). Finally, the* 11-E3* mAb had a third staining pattern with low-level binding to IgG^int^ cells but did not bind to IgG^hi^ cell population.

**Figure 5:**
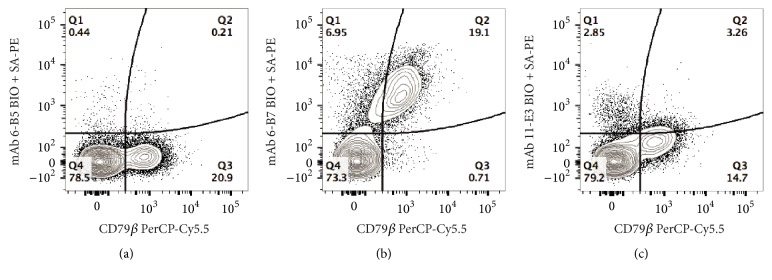
Surface staining with heavy chain reactive monoclonal antibodies. Biotinylated mouse anti-ferret Ig mAb were used in combination with anti-CD79*β* to evaluate surface staining of ferret B cells. Binding of biotinylated (a)* 6-B5*, (b)* 6-B7*, or (c)* 11-E3* was revealed by secondary staining with phycoerythrin conjugated streptavidin (SA-PE). Frequency of CD79*β*^neg/pos^ ferret PBMC that costained with the mouse anti-ferret Ig mAb are indicated. Data shown were generated using a single ferret and are representative of two or more independent experiments that yielded similar results.

As a next step to further characterize the reagents, directly labeled M*α*F-Ig mAb were used to stain ferret PBMC in combination with anti-CD79*β* (Figure 5). Based on previously observed western blot and flow cytometric staining patterns (Figures 3 and 4), the M*α*F-Ig mAb were segregated into two classifications. The first group of M*α*F-Ig mAb comprised* 6-B5*,* 6-B7*, and* 11-E3* and these mAb had a reactivity profile consistent with Ig heavy chain specificity. Despite evidence of reactivity with IgG^hi^ cells in the indirect screen (data not shown), biotinylated* 6-B5* did not stain CD79*β*^+^ ferret B cells (Figure 5(a)). By contrast, biotinylated* 6-B7* stained almost all of the CD79*β*^+^ cell population (Figure 5(b)). In addition,* 11-E3* stained the entire CD79*β*^+^ cell population with a low-level of fluorescence, as well as a small population of CD79*β*^neg^ cells (Figure 5(c)). Similar results were also observed using these same mAb following DyLight conjugation (data not shown).

**Figure 6:**
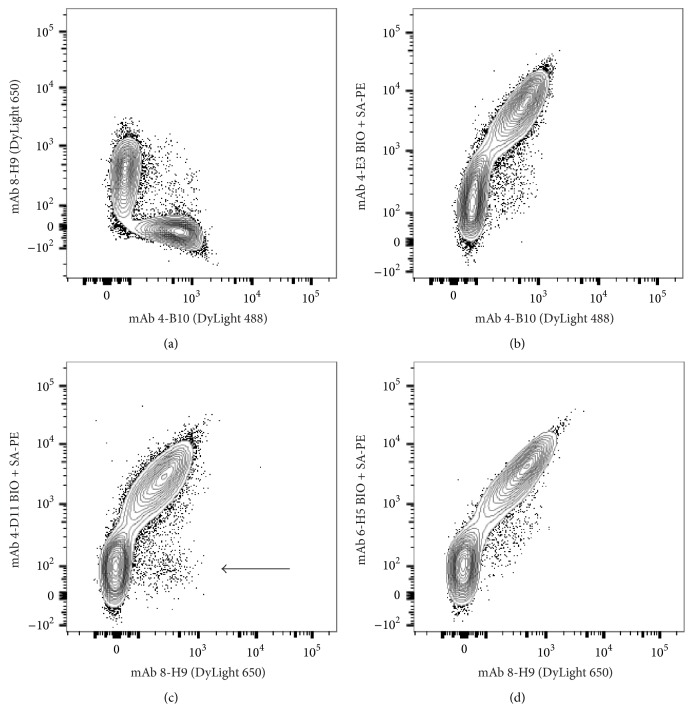
Characterization of light chain reactive monoclonal antibodies. Mouse anti-ferret Ig mAb were used in combination for surface staining of ferret PBMC. Plots are pregated on B cell receptor expressing cells (CD79*β*^+^) as shown in Supplementary Materials. (a) Dual staining of ferret B cells with* 4-B10* and* 8-H9*. (b) Dual staining of ferret B cells with* 4-B10* and* 4-E3*. (c) Dual staining of ferret B cells with* 8-H9* and* 4-D11*. Arrow indicates population of 8-H9^+^ B cells that lack costaining with* 4-D11*. (d) Dual staining of ferret B cells with* 8-H9* and* 6-H5*. Data are representative of two or more independent experiments comprising *n* ≥ 6 individual ferrets.

The second group of M*α*F-Ig mAb (*4-B10* and* 8-H9*) exhibited a reactivity profile consistent with light chain specificity. However, double-labeling CD 79*β*^+^ ferret PBMC with these mAb revealed staining of distinct populations of cells (Figure 6(a)). Moreover, nearly the entire CD79*β*^+^ population stained positive with either* 4-B10* or* 8-H9*, with few cells reacting with both mAb. Collectively, these findings suggested that* 4-B10* and* 8-H9* define distinct populations of ferret B cells on the basis of light chain expression.

The remaining M*α*F-Ig mAb (*4-E3*,* 4-D11*, and* 6-H5*) also had light chain reactivity. These mAb were categorized on the basis of costaining with* 4-B10* or* 8-H9* single-positive ferret B cells (Figures 6(b)–6(d)). Using a multilabeling approach,* 4-E3* was found to costain with nearly the entire 4-B10^pos^ population, while simultaneously not labeling 8-H9^pos^ cells (Figure 6(b) and data not shown). Similarly, 8-H9^pos^ cells were labeled by* 4-D11* or* 6-H5*, and these mAb exhibited minimal reactivity with 4-B10^pos^ cells (Figures 6(c) and 6(d)). Of note, we routinely observed a small population of 8-H9^pos^ cells that were not stained by* 4-D11* (Figure 6(c)). Collectively, these data indicate that* 4-B10* and* 4-E3* recognize a common light chain protein that is distinct from the light chain recognized by the other mouse anti-ferret light chain (M*α*F-IgL) mAb (*8-H9*,* 4-D11*, and* 6-H5*).

### 3.7. Identification of Overlapping Epitope Recognition through Competitive Binding ELISA

**Figure 7:**
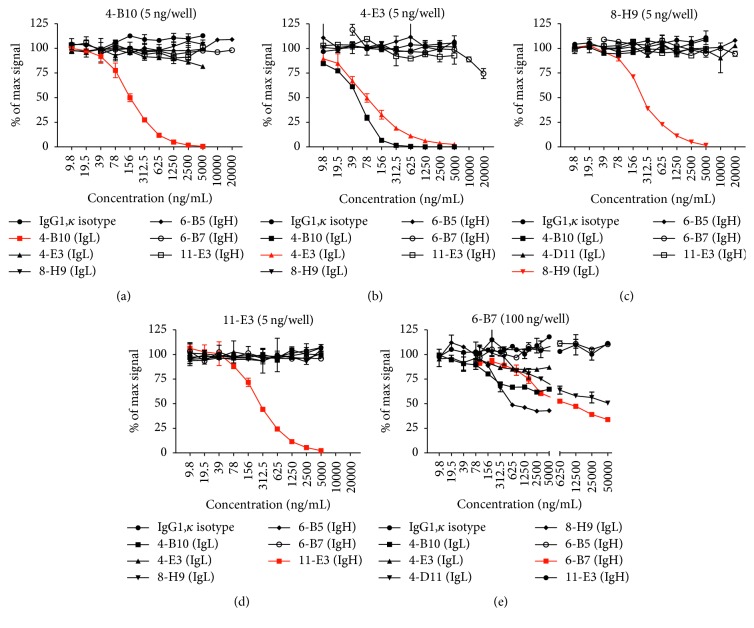
Mapping of monoclonal antibodies through competitive ELISA. Inhibition of biotinylated mouse anti-ferret Ig mAb binding to purified ferret Ig was performed to identify overlapping epitope recognition. Competitive inhibition of (a)* 4-B10*, (b)* 4-E3*, (c)* 8-H9*, (d)* 11-E3*, and (e)* 6-B7* were performed using the indicated concentration of biotinylated mAb. The *x*-axis indicates concentration of unlabeled mouse anti-ferret Ig mAb competitor, and the homologous unlabeled mAb are depicted in red. Percent of maximal binding by the respective biotinylated mAb is plotted ± SD of duplicates. Data in (a)–(e) are representative of two independent experiments.

To further characterize whether the M*α*F-Ig mAb were recognizing overlapping or distinct epitopes on ferret Ig, competitive binding assays were performed. Specifically, unlabeled M*α*F-Ig mAb were used as competitors and evaluated for their ability to inhibit binding of biotinylated mAb (*4-B10*,* 4-E3*,* 6-B7*,* 8-H9*, and* 11-E3*) to the purified ferret Ig antigen. Consistent with the double-labeling FCM studies in which* 4-B10* and* 8-H9* recognized distinct populations of ferret PBMC (Figure 6(a)), these M*α*F-Ig mAb did not exhibit inhibition of each other in the competitive binding ELISA (Figures 7(a) and 7(c)). In addition, despite recognition of the same population as assessed by double-labeling FCM (Figure 6(b)),* 4-E3* exhibited only subtle inhibition of* 4-B10* at the highest concentration tested (5 *µ*g/mL) (Figure 7(a)). In stark contrast,* 4-B10* competed for binding with biotinylated* 4-E3* (Figure 7(b)). Moreover,* 4-B10* exhibited superior competition with biotinylated* 4-E3* relative to the homologous unlabeled competitor. No competition of biotinylated* 8-H9* was observed by any M*α*F-Ig mAb tested. Strikingly, even unlabeled* 4-D11* at 20 *µ*g/mL failed to compete with* 8-H9* despite these mAb labeling a common population of ferret PBMC by FCM (Figures 6(c) and 7(c)). Similarly, none of the M*α*F-Ig mAb exhibited competition with biotinylated* 11-E3* (Figure 7(d)). However, a number of M*α*F-IgL mAb were found to compete with biotinylated* 6-B7*, despite assignment of this mAb as heavy chain specific (Figure 7(e)). Specifically, both unlabeled* 4-B10* and* 8-H9* demonstrate definitive competition with biotinylated* 6-B7* (Figure 7(e)). Moreover,* 4-E3* and* 4-D11* (only at high concentrations) were also capable of inhibiting binding of biotinylated* 6-B7* to the ferret Ig antigen, albeit to a less extent relative to* 4-B10* and* 8-H9*.

### 3.8. Determination of Light Chain Specificity

Although the individual M*α*F-IgL mAb were already segregated into two distinct groups on the basis of light chain reactivity, their precise specificity remained undefined. To determine which group of M*α*F-IgL mAb was specific for the Ig*κ* chain, a fluorescently labeled bacterial protein with specificity for Ig*κ* chains from a variety of species [28, 29], Protein L, was used to label ferret B cells. Approximately 25% of CD79*β*^+^ ferret B cells stained brightly with the fluorescently conjugated Protein L reagent (Figure 7(a)) and these cells were putatively assigned as Ig*κ*-expressing B cells. Next, these CD79*β*^+^ Protein L^hi^ cells were evaluated for costaining with* 4-B10* or* 8-H9*, which defined distinct subsets of ferret B cells. The majority (~80%) of CD79*β*^+^ Protein L^hi^ cells were costained with* 4-B10* (Figure 7(b)) and indicated that* 4-B10* was specific for ferret Ig*κ*. Conversely, this result indicated that* 8-H9* was specific for Ig*λ*. Using these assignments, the distribution of Ig*κ* and Ig*λ* expression by circulating CD79*β*^+^ ferret B cells was determined (Figure 7(c)).

## 4. Discussion

In this study, eight individual mAb with specificity for ferret Ig were characterized (Table 1). Five of these mAb had specificity for ferret Ig light chain, while the remaining three mAb did not recognize a distinct surface expressed heavy chain. Additionally, we generated a mAb* (6-B7)* that yielded surface staining of nearly all surface BCR positive cells (*i.e.*, pan ferret Ig). Collectively, these M*α*F-Ig mAb offer advancement in ferret Ig detection compared to the existing portfolio of polyclonal antisera reagents. Moreover, several of these mAb (*4-B10*,* 8-H9*, and* 11-E3*) are likely to be applicable to a wide array of immunologic assays, including the identification of antibody-secreting cells by FCM.

Pooled serum was chosen as the source of material for subsequent purification of ferret Ig because both IgM and IgG antibody classes were abundant and this biological fluid was readily available [26]. Moreover, the purified ferret Ig preparation had the potential to elicit mAb with specificity for the heavy chain (Ig*µ* or Ig*γ*) and the light chain (Ig*κ* or Ig*λ*) using a single antigen. However, due to the complexity of the ferret Ig antigen, antigen-specific binding in the ELISA format was insufficient to determine the precise specificity of the respective M*α*F-Ig mAb (Figure 2). To this end, additional screening approaches, such as FCM, western blot and competitive binding assays, were necessary to further define the specificity and categorized the individual M*α*F-Ig mAb.

Implementation of an indirect FCM screening method during the initial characterization enabled identification of M*α*F-Ig mAb that recognized epitopes present on ferret leukocytes. Moreover, incorporation of the G*α*F-Ig*γ* antisera in this screen enabled resolution of ferret PBMC into distinct populations on the basis of staining intensity, and provided insight into the specificity of the respective M*α*F-Ig mAb (Figure 4 and data not shown). Specifically, each of M*α*F-IgL mAb exhibited a similar staining pattern in which approximately half of the sIgG^hi^ population was intensely labeled by the respective mAb. Moreover, these M*α*F-IgL mAb also labeled a small subset of the sIgG^int^ population at a similarly intense level, while the remainder of the sIgG^int^ population exhibited a low-level of staining (Figures 4(c) and 4(d) and data not shown). This staining pattern indicated that surface Ig^+^ B cells constituted a fraction of the sIgG^int^ population and was consistent with the observation that the G*α*F-Ig*γ* antisera as well as other commercially available polyclonal anti-ferret Ig antisera fail to label all B cells (data not shown and [14]). Additionally, this staining pattern implied that myeloid cells constituted a portion of the sIgG^int^ population and that IgG acquired via FcR was bound in such manner that the light chain was accessible to the respective M*α*F-IgL mAb. Furthermore, segregation of the IgG^int^ and sIgG^hi^ populations in the FCM screen also facilitated perception of the distinct binding specificities exhibited by the heavy chain reactive mAb* 6-B7* and* 11-E3* on the basis of their differential reactivity profile with the sIgG^hi^ population (Figures 4(e) and 4(f)). Lastly, in the absence of the apparent disappearance of the sIgG^hi^ population that resulted following pretreatment of ferret PBMC with* 6-B5* (data not shown), this M*α*F-Ig mAb would not have been characterized further.

Despite the perceived reactivity of* 6-B5* in the antigen-specific capture ELISA and indirect FCM screening during the initial characterization, definitive staining of ferret B cells using this mAb, when directly coupled, was not observed (Figure 5(a) and data not shown). It is likely that biotinylation of* 6-B5* was inefficient, rather than that conjugation destroyed the binding specificity of this mAb. Specifically, both unlabeled and biotinylated* 6-B5* exhibited comparable binding to ferret Ig in an ELISA when using a goat anti-mouse IgG1 secondary reagent (data not shown). By contrast, the same biotinylated* 6-B5* exhibited a near absence of reactivity against ferret Ig when streptavidin was used to reveal binding. Consequently, it will be necessary to explore alternative chemistries for conjugation of* 6-B5* to fully realize the utility of this mAb for flow cytometric applications.

The competitive binding ELISA contributed to the characterization of the respective M*α*F-Ig mAb. First, the data generated using this approach served to reaffirm prior observations, such as the distinction between* 4-B10* and* 8-H9* determined by double-labeling FCM. Second, the competitive binding and antigen-specific capture ELISA data were in agreement and reflected differences in functional affinity by several of the M*α*F-Ig mAb. Most notably,* 4-B10* exhibited stronger avidity for the ferret Ig antigen relative to* 4-E3* (Figure 2) and also demonstrated superior competition of biotinylated* 4-E3* compared to the homologous mAb (Figure 7(b)). Thirdly, the competitive binding ELISA also provided additional insight into the epitopes recognized by the respective M*α*F-Ig mAb. While it was already presumed that the M*α*F-IgL mAb were targeting the constant region of kappa or lambda light chain based on their FCM staining patterns (Figure 6), it remained unclear if these anti-kappa or anti-lambda mAb were targeting distinct or overlapping epitopes. On the basis of competitive inhibition, it is likely that* 4-B10* and* 4-E3* are recognizing similar, but not completely overlapping epitopes on the kappa constant region since the inhibition was unidirectional (Figures 7(a) and 7(b)). By contrast,* 4-D11* failed to inhibit binding of biotinylated* 8-H9* despite the use of an elevated concentration (20 *µ*g/mL), suggesting that these mAb recognize distinct epitopes on the lambda constant region. However, it remains plausible that the large difference in functional affinity between these mAb contributed to the lack of competition (Figures 2 and 7(c)). Additionally, biotinylated* 4-D11* failed to produce a sufficiently high binding signal against the ferret Ig antigen to enable evaluation of reciprocal inhibition by* 8-H9* (data not shown). Similar to* 8-H9*, no inhibition of biotinylated* 11-E3* was observed using any of the M*α*F-Ig mAb tested, further supporting the notion that this mAb recognizes a distinct epitope (Figure 7(d)). Surprisingly, several of the M*α*F-IgL mAb were capable of inhibiting binding of biotinylated* 6-B7* to the ferret Ig antigen. This was unexpected since* 6-B7* was categorized as heavy chain reactive based on the observed FCM staining pattern (Figure 5(c)). However, the ability of* 4-B10* and* 8-H9*, as well as* 4-E3* and* 4-D11* to a less extent, to compete with* 6-B7* supports the conclusions that* 6-B7* is recognizing an epitope that is restricted to the heavy chain. Moreover, these data suggest that* 6-B7* recognizes an epitope on the heavy chain of ferret Ig that is proximal to either the kappa or lambda constant region. To this end, we hypothesize that* 6-B7* would maintain reactivity with a Fab fragment of ferret Ig.

In spite of strong reactivity with purified ferret Ig by ELISA (Figure 2) and the Ig*γ* chain by western blot (Figure 3(d)), intense staining of ferret B cells using directly labeled* 11-E3* was not observed (Figure 5(c)). However, low-level staining of the entire CD79*β*^+^ B cell population, as well as a small population of non-B cells in bulk ferret PBMC, was observed using* 11-E3*. Collectively, these observations indicate that the epitope recognized by* 11-E3* may be restricted to secreted ferret IgG. Further experimentation will be necessary to resolve the utility of* 11-E3* for studying ferret B cells and derived Ig.

**Figure 8:**
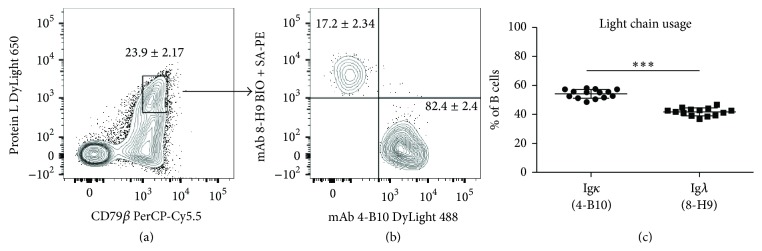
Designation of light chain reactive monoclonal antibody specificity. Mouse anti-ferret Ig mAb (*4-B10* and* 8-H9*) were used in combination with anti-CD79*β* and DyLight 650 conjugated Protein L to assign mAb reactivity with kappa light chain expressing B cells. (a) Representative surface staining of ferret PBMC with anti-CD79*β* and Protein L. Kappa light chain expressing B cells were defined as CD79*β*^+^ cells that stained intensely with DyLight 650 conjugated Protein L and were gated as shown in (a). The mean frequency of total B cells ± SD that stained intensely with Protein L is indicated. (b) Representative costaining of kappa light chain expressing B cells with* 4-B10* or* 8-H9*. The mean frequency ± SD of total kappa B cells exhibiting costaining with* 4-B10* or* 8-H9* is indicated. Data in (a) and (b) are combined from two independent experiments (*n* = 6). (c) Distribution of kappa or lambda light chain expression by total B cells (CD79*β*^+^) was determined on the basis of costaining with* 4-10* or* 8-H9*. Data in (c) are combined from three independent experiments (*n* = 14). Statistical significance was determined using a paired Student's *t*-test. ^*∗∗∗*^*p* < 0.001.

The assignment of lambda light chain specificity to* 8-H9* was based upon several independent observations. First, the near absolute segregation between populations of 4-B10^+^ and 8-H9^+^ ferret B cells indicated that these mAb recognized fundamentally distinct light chains (Figure 6(a)). Of note, this observation also indicates that allelic exclusion of dual light chain expression is largely intact in the ferret model [30]. Secondly, 8-H9^+^ ferret B cells were severely underrepresented in the population of B cells that bound the kappa-specific Protein L reagent at high levels (Figures 8(a) and 8(b)). While there was a population of 8-H9^+^ B cells in this gate (~20%), it is plausible that these Ig*λ*^+^ B cells acquired labeling with Protein L either due to inherent BCR specificity or through the association of secreted Ig*κ* with surface expressed FcR [31, 32]. In addition, within the total B cell population, the frequency of 8-H9^+^ B cells detected from multiple independent ferrets closely resembled the distribution of Ig*λ*^+^ B cells observed in humans [33].

While a draft sequence of the ferret genome is currently available [34], the Ig loci have not been annotated. Based on the findings presented in this report, we hypothesize that the ferret Ig*λ* locus more closely resembles the human Ig*λ* locus than the mouse Ig*λ* loci due to the increase proportion of B cells that express a lambda light chain (Figure 8(c)). In laboratory mice, the Ig*λ* locus is comprised of 3 functional V*λ* gene elements that rearrange with associated J*λ*-C*λ* gene elements [35]. Additionally, in mice the prevalence of serum immunoglobulin utilizing a lambda light chain is severely reduced relative to immunoglobulin utilizing a kappa light chain [36]. By contrast, the human Ig*λ* locus is more complex and encodes greater than 35 V*λ* genes belonging to 10 subgroups [37]. Moreover, the distribution of kappa and lambda immunoglobulin is more balanced in human serum [38]. Collectively, these findings provide the first evidence that lambda light chain usage in ferrets closely resembles that seen in humans and further supports the use of ferrets for studying the antibody response elicited by influenza virus infection or influenza vaccination.

To more accurately mimic influenza infection and vaccination in humans, ferrets can be infected with various influenza viruses from different subtypes to establish a preimmune state in the animal. This model is useful to study both inactivated and live attenuated influenza vaccine (LAIV) candidates. In young children, an IgA recall response occurred following a second administration of LAIV [39]. However, ferrets previously infected with LAIV had a robust IgG antibody-secreting cell (ASC) response in the absence of an accompanying IgA ASC population following experimental challenge with seasonal influenza [40]. While it remains plausible that the immune response elicited by influenza infection of preimmune ferrets was distinct from that observed in young children following LAIV inoculation, it is more likely that the discrepancy between these two models originates from the use of polyclonal anti-ferret Ig detection reagents that possess an inherent lack of heavy chain specificity. This apparent lack of heavy chain specificity was observed using a variety of polyclonal goat anti-ferret Ig reagents (Supplementary Materials and data not shown). While mAb with specificity for discrete ferret Ig heavy chain determinants were unfortunately not generated in this study, this example serves as motivation for the continued development and characterization of ferret Ig-specific reagents.

In addition, a polyclonal goat anti-ferret Ig reagent with specificity for IgA, IgG, and IgM was used to identify ferret B cells in the context of influenza infection using FCM [14]. In this study, the polyclonal goat anti-ferret Ig (A, G, and M) failed to label a substantial population of ferret B cells that exhibited intracellular staining with the anti-CD79*α* mAb (clone HM47). The M*α*F-Ig mAb reported here are an improvement over existing reagents and enable the identification of nearly all surface Ig^+^ ferret B cells (Figure 5(b)) and discrimination between Ig*κ* and Ig*λ*-expressing cells (Figure 6(a)).

We envision that several of the M*α*F-Ig mAb generated in this study will have applications in basic science and veterinary medicine. Due to their strong affinity for purified ferret Ig and reactivity with denatured heavy (Ig*γ*) or light chain proteins,* 4-B10*,* 8-H9*, and* 11-E3* may prove to be most useful. Specifically, these M*α*F-Ig mAb should enable the identification of antibody-secreting cells by FCM. Additionally, these mAb are likely to have utility as secondary detection reagents in an array of assays such as ELISA, ELISPOT, and western blot. Implementation of these mAb in a variety of immunologic assays will also contribute towards the assessment of the next-generation of broadly-reactive influenza vaccines in this highly relevant model. In summary, the generation of these M*α*F-Ig mAb is an improvement over existing reagents available to immunologists and opens the door to more sophisticated study of ferret B cells.

## Supplementary Material

The supplementary material offers data supporting the lack of heavy chain specificity observed using commercially available anti-ferret immunoglobulin antiserum. Additionally, the mouse immunization scheme and characterization of the polyclonal serum antibody response against the ferret Ig antigen are detailed.

## Figures and Tables

**Table 1:** Summary of M*α*F-Ig mAb.

mAb	Ig subclass	V_H_^a^	V_L_^a^	Specificity
4-B10	IgG1	IGHV4-1	IGKV6-13	Light chain (Ig*κ*)^c,d^
4-D11	IgG1	IGHV2-3	IGKV16-104	Light chain (Ig*λ*)^d^
4-E3^b^	IgG1	IGHV1-20 or IGHV1-37	IGKV3-12	Light chain (Ig*κ*)^d^
6-B5	IgG1	IGHV5-6	IGKV19-93	Ferret Ig
6-B7	IgG1	IGHV5-6	ND^e,f^	Heavy chain (IgH)^g^
6-H5	IgG1	IGHV5-17	IGKV5-48	Light chain (Ig*λ*)^d^
8-H9	IgG1	IGHV14-3	IGKV6-32	Light chain (Ig*λ*)^c,d^
11-E3	IgG1	IGHV1S81	IGKV6-17	Heavy chain (Ig*γ*)^c^

a: identified using IMGT V-Quest (see [21]). b: unable to unambiguously assign IGHV. c: determined by western blot. d: determined by flow cytometry. e: not determined. f: negative for intracellular Ig*κ* by flow cytometry. g: determined by competitive ELISA.
